# Molecular Evolution of Human H1N1 and H3N2 Influenza A Virus in Thailand, 2006–2009

**DOI:** 10.1371/journal.pone.0009717

**Published:** 2010-03-16

**Authors:** Kamol Suwannakarn, Thaweesak Chieochansin, Chitima Thongmee, Jarika Makkoch, Kesmanee Praianantathavorn, Apiradee Theamboonlers, Srinand Sreevatsan, Yong Poovorawan

**Affiliations:** 1 Center of Excellence in Clinical Virology, Faculty of Medicine, Chulalongkorn University, Bangkok, Thailand; 2 Departments of Veterinary Population Medicine and Veterinary and Biomedical Sciences, University of Minnesota, Saint Paul, Minnesota, United States of America; Institute of Infectious Disease and Molecular Medicine, South Africa

## Abstract

**Background:**

Annual seasonal influenza outbreaks are associated with high morbidity and mortality.

**Objective:**

To index and document evolutionary changes among influenza A H1N1 and H3N2 viruses isolated from Thailand during 2006–2009, using complete genome sequences.

**Methods:**

Nasopharyngeal aspirates were collected from patients diagnosed with respiratory illness in Thailand during 2006–2009. All samples were screened for Influenza A virus. A total of 13 H1N1 and 21 H3N2 were confirmed and whole genome sequenced for the evolutionary analysis using standard phylogenetic approaches.

**Results:**

Phylogenetic analysis of HA revealed a clear diversification of seasonal from vaccine strain lineages. H3N2 seasonal clusters were closely related to the WHO recommended vaccine strains in each season. Most H1N1 isolates could be differentiated into 3 lineages. The A/Brisbane/59/2007 lineage, a vaccine strain for H1N1 since 2008, is closely related with the H1N1 subtypes circulating in 2009. HA sequences were conserved at the receptor-binding site. Amino acid variations in the antigenic site resulted in a possible N-linked glycosylation motif. Recent H3N2 isolates had higher genetic variations compared to H1N1 isolates. Most substitutions in the NP protein were clustered in the T-cell recognition domains.

**Conclusion:**

In this study we performed evolutionary genetic analysis of influenza A viruses in Thailand between 2006–2009. Although the current vaccine strain is efficient for controlling the circulating outbreak subtypes, surveillance is necessary to provide unambiguous information on emergent viruses. In summary, the findings of this study contribute the understanding of evolution in influenza A viruses in humans and is useful for routine surveillance and vaccine strain selection.

## Introduction

Influenza A viruses are negative-strand RNA viruses of the family *Orthomyxoviridae* which can be divided into subtypes based on the antigenic properties of the surface glycoproteins, hemagglutinin (HA) and neuraminidase (NA) [1]. Every year influenza A virus causes infection with varying severity depending on host acquired immunity against that particular virus strain. Influenza epidemics are associated with above average annual mortality levels, causing 10,000 to 15,000 deaths. Occasionally global pandemics of influenza occur, infecting 20% to 40% of the population in a single year and dramatically raising death rates. Three such major global pandemics caused by novel antigenic variants of influenza viruses have affected the human population: in 1918 (H1N1 subtype), in 1957 (H2N2), and in 1968 (H3N2) resulting in millions of deaths [2], [3]. The recent circulation of highly pathogenic avian H5N1 viruses in Asia since 2003 has caused human fatalities [4]. The novel H1N1 influenza virus that emerged in humans in Mexico in early 2009 and spread globally in the human population has been declared a pandemic strain.

Two forms of the 16 possible HA subtypes (H1 and H3), and two of the nine possible NA subtypes (N1 and N2) are circulating in man. H3N2 and H1N1 influenza A viruses have co-circulated in the human population since the re-emergence of H1N1 in 1977 increasing the possibility of genetic reassortment. The prevalence of different subtype combinations may vary from season to season. Subtype H3N2 has constituted the predominant influenza A strain during the last 20 years, with the exception of the 1988–1989 and 2000–2001 seasons when H1N1 infections were more prevalent [5]. The surface glycoprotein HA is under selective pressure to undergo mutation in order to evade the host's immune system [6]. Antibodies against the HA protein inhibit receptor binding and are very effective at preventing re-infection with the same strain. However, HA can change to evade previously acquired immunity either by antigenic drift, whereby mutations of the currently circulating HA gene disrupt antibody binding, or by antigenic shift, with the virus acquiring an HA of a new subtype by re-assortment of one or more gene segments. The WHO has published recommendations on the composition of influenza vaccine for the northern and southern hemispheres. For the northern hemisphere, the WHO has issued the recommendation for strains to be included in the trivalent vaccine for the next season based on epidemiological data and genetic analyses of circulating strains.

The ability to predict emergence of circulating influenza strains for subsequent annual vaccine development has become vital. Comparisons between antigenic differences and phylogenetic analysis are essential to further the understanding of emergence of multiple lineages of influenza virus variants. Therefore, the aim of this study is to elucidate the evolution of influenza A H1N1 and H3N2 isolates from Thailand over a four years time period early 2006 to 2009. Understanding of viral genome content and its evolution is experted to aid in predicting the emergence of new variant strains during the following season and defining the vaccine composition.

## Results

Specimens were collected from patients diagnosed with respiratory illness in Bangkok, Thailand from 2006 to 2009. Clinical specimens were screened for influenza A virus by multiplex real-time PCR and then subtyped as H1 or H3 with specific primers. Of the influenza A virus positive samples, 21H3N2 and 13 H1N1 samples were selected to represent each time-point, respectively. The HA and NA sequences of some strains isolated in 2006 to 2007 have been previously reported [13]. The details are shown in **Table 1**. (accession numbers are provided in supporting information Table S1).

**Table 1:** The details of the specimens.

Subtype	Strain	Date	Sex	Age	Seasonal cluster
H1N1	A/Thailand/CU32/2006	13-04-2006	Female	3 years	A/NewCaledonia/20/1999 lineage
	A/Thailand/CU44/2006	13-05-2006	Female	11 years	A/Solomon Island/3/2006 lineage
	A/Thailand/CU51/2006	30-05-2006	Male	2 years	A/Solomon Island/3/2006 lineage
	A/Thailand/CU68/2006	26-06-2006	Female	8 months	A/Solomon Island/3/2006 lineage
	A/Thailand/CU88/2006	25-07-2006	Female	5 months	A/Solomon Island/3/2006 lineage
	A/Thailand/CU-B42/2009	16-06-2009	Male	39 years	A/Brisbane/59/2007 like lineage
	A/Thailand/CU-H17/2009	18-06-2009	Female	34 years	A/Brisbane/59/2007 like lineage
	A/Thailand/CU-B97/2009	22-06-2009	Male	22 years	A/Brisbane/59/2007 like lineage
	A/Thailand/CU-B267/2009	02-07-2009	Female	33 years	A/Brisbane/59/2007 like lineage
	A/Thailand/CU-B589/2009	10-07-2009	Male	43 years	A/Brisbane/59/2007 like lineage
	A/Thailand/CU-B685/2009	12-07-2009	Female	45 years	A/Brisbane/59/2007 like lineage
	A/Thailand/CU-H223/2009	17-07-2009	Female	36 years	A/Brisbane/59/2007 like lineage
	A/Thailand/CU-H565/2009	02-09-2009	Female	60 years	A/Brisbane/59/2007 like lineage
H3N2	A/Thailand/CU23/2006	22-03-2006	Female	1 year	2005–2006 season
	A/Thailand/CU46/2006	18-05-2006	Male	11 months	2005–2006 season
	A/Thailand/CU228/2006	11-10-2006	Male	2 years	2006–2007 season
	A/Thailand/CU231/2006	14-11-2006	Male	1 year	2006–2007 season
	A/Thailand/CU260/2006	27-12-2006	Female	1 year	2006–2007 season
	A/Thailand/CU280/2007	26-01-2007	Female	2 years	2006–2007 season
	A/Thailand/CU282/2007	02-02-2007	Male	3 years	2006–2007 season
	A/Thailand/CU1101/2008	25-02-2008	Female	3 years	2007–2008 season
	A/Thailand/CU1102/2008	20-03-2008	Male	3 years	2007–2008 season
	A/Thailand/CU1103/2008	24-03-2008	Male	10 years	2007–2008 season
	A/Thailand/CU356/2008	25-03-2008	Male	11 months	2007–2008 season
	A/Thailand/CU370/2008	16-06-2008	Male	5 months	2007–2008 season
	A/Thailand/CU379/2008	04-07-2008	Female	9 months	2007–2008 season
	A/Thailand/CU-B4/2009	13-06-2009	Female	5 years	2009 season
	A/Thailand/CU-H16/2009	17-06-2009	Female	22 years	2009 season
	A/Thailand/CU-B106/2009	22-06-2009	Male	7 years	2009 season
	A/Thailand/CU-B110/2009	23-062009	Male	8 years	2009 season
	A/Thailand/CU-B590/2009	10-07-2009	Female	40 years	2009 season
	A/Thailand/CU-B657/2009	11-07-2009	Female	29 years	2009 season
	A/Thailand/CU-B1672/2009	30-10-2009	Female	22 years	2009 season
	A/Thailand/CU-B1697/2009	10-11-2009	Female	59 years	2009 season

**Figure 1:**
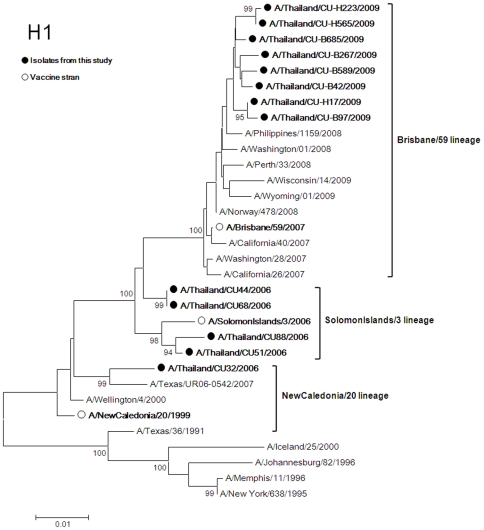
Phylogenetic tree of H1. The trees were constructed by the Neighbor-Joining method with bootstrap support by 1,000 replicates. (• represents isolates from this project and ο represents vaccine strain).

Five H1N1 isolates from the 2006 season and eight isolates from 2009 were compared with the vaccine strains for phylogenetic associations. H1 phylogeny showed that the isolates clustered in 3 distinct lineages (**Fig. 1**). First, the HA of A/Thailand/CU32/2006 was closely related to the A/NewCaledonia/20/1999 lineage (vaccine strain for 2007 Southern Hemisphere) by 97.84% and 98.21% based on nucleotide and amino acid similarity, respectively. Second, four isolates from 2006 were closely related to the A/Solomon Island/3/2006 lineage (vaccine strain for 2007–2008 Northern Hemisphere). The average percent nucleotide and amino acid identities of HA to this vaccine strain are 98.67% and 98.25%, respectively. Last, the phylogenetic tree of H1 showed that five isolates from 2009 were closely related with A/Brisbane/59/2007 like lineage (vaccine strain for 2008–2009 Northern Hemisphere, 2009 Southern Hemisphere and 2009–2010 Northern Hemisphere). These similarities between isolates and vaccine strain lineage were slightly different at 99.25% and 99.24% based on nucleotide and amino acid sequences, respectively.

**Figure 2:**
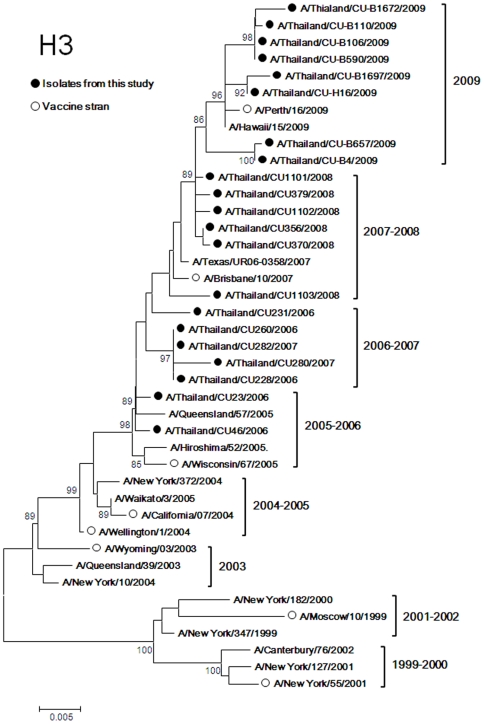
Phylogenetic tree of H3. The trees were constructed by the Neighbor-Joining method with bootstrap support by 1,000 replicates. (• represents isolates from this project and ο represents vaccine strain).

Twenty one HA sequences of H3N2 isolates were compared with vaccine strains (**Fig. 2**). The results showed that H3 nucleotide sequences formed seasonal phylogenetic clusters representative for the 2005–2006, 2006–2007, 2007–2008 and 2009 seasons. The H3 nucleotide sequences of the 2005–2006 season were closely related to the A/Wisconsin/67/2005 like lineage (vaccine strain for 2006–2007 Northern Hemisphere, 2007 Southern Hemisphere, 2007–2008 Northern Hemisphere). The average nucleotide similarity of HA to this vaccine strain is 99.49% and 98.53% based on amino acids sequences. The 2006–2007 strains were distinct from the 2005–2006 strains. The average nucleotide similarities among the 2006–2007 and 2005–2006 strains were 99.029% (98.88% based on amino acids) and similarities of the 2006–2007 strain to the A/Wisconsin67/2005 like lineage decreased to 98.985% (97.64% based on amino acids). During the following season, 2007–2008, the H3 nucleotide sequence had drifted more towards A/Brisbane/10/2007 like lineage (vaccine strain for 2008 Southern Hemisphere, 2008–2009 Northern Hemisphere, 2009 Southern Hemisphere and 2009–20010 Northern Hemisphere) (99.392% and 99.24% similarity based on nucleotide and amino acids sequences, respectively).

The H3 nucleotide sequences of the 2009 virus isolates had drifted from the the 2007–2008 strain. These strains were unique to the 2009 season. The nucleotide similarities between the 2009 and 2007–2008 strains were 98.98% (98.86% based on amino acids); between the 2009 strains and the A/Brisbane/10/2007 like lineage they decreased to 98.83% (98.62% based on amino acids). The viruses from this season were closely related to the A/Perth/16/2009-like lineage (vaccine strain for 2010 Southern Hemisphere). The nucleotide identities between the 2009 strains and the A/Perth/16/2009-like lineage were 99.64% and 99.36% based on amino acids.

**Figure 3:**
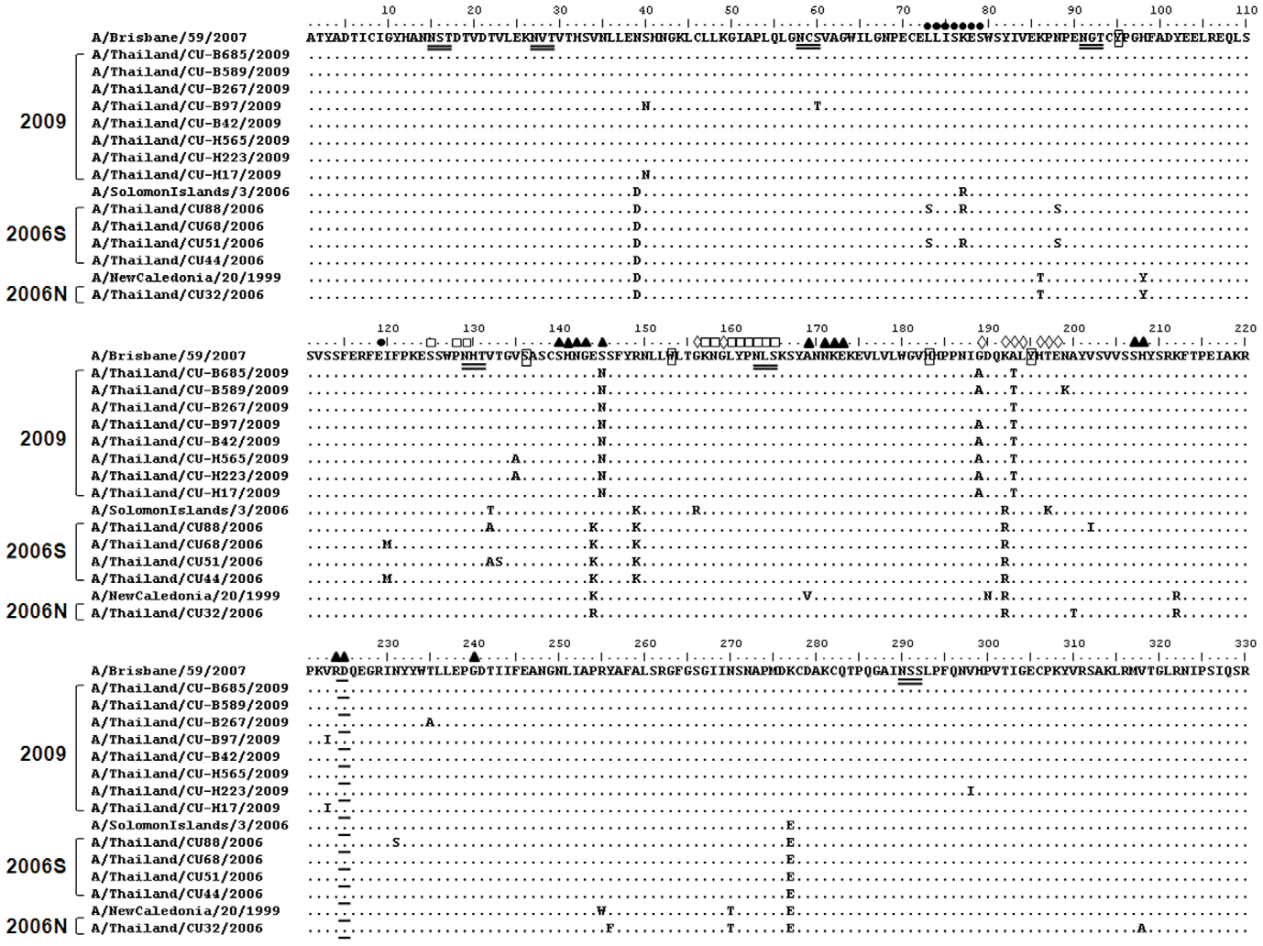
Amino acid comparison between HAl domains of H1N1 isolates and vaccine strains. Dots represent amino acids similar to the consensus. The conserved amino acid residues at the receptor-binding site are shown as small rectangles. Alternative amino acids for sialic acid linkages of HA are underlined. The amino acid residues mapped at previously defined antigenic sites are shown as follows: site Sa (□), site Sb (◊), site Ca (▴), and site Cb (•).The the potential N-glycosylation sites, with threshold value of >0.5, are double-underlined. 2009 represents the circulating strain in 2009 which belong to A/Brisbane/59/2007-like lineage. 2006S represents the circulating strain in 2006 which belong to A/Solomon Island/3/2006-like lineage. 2006N represents the circulating strain in 2006 which belong to A/New Caledonia/20/1999-like lineage.

**Figure 4:**
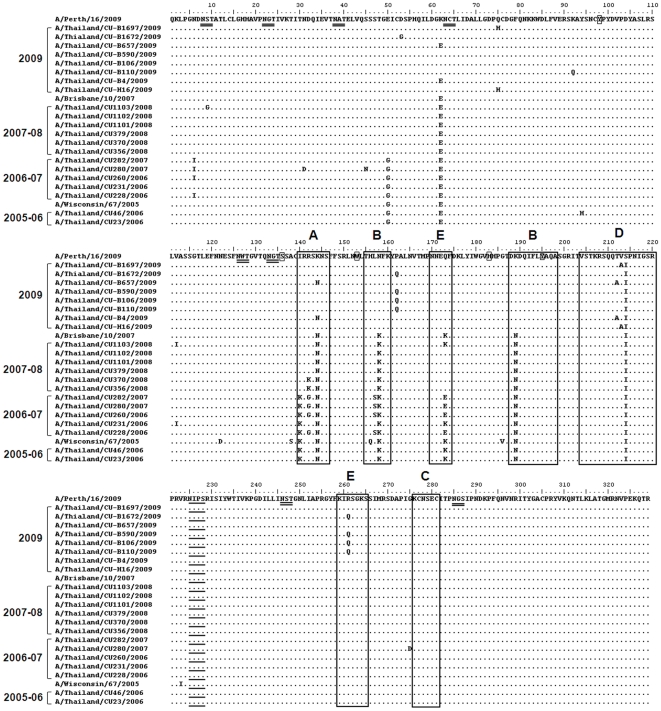
Amino acid comparison between HAl domains of H3N2 isolates and vaccine strains. Dots represent amino acids similar to the consensus. The conserved amino acid residues at the receptor-binding site are shown as small rectangles. Alternative amino acids for sialic acid linkages of HA are underlined. The amino acid residues mapped at previously defined antigenic sites A–E are shown as large rectangles. The the potential N-glycosylation sites, with threshold value of >0.5, are double-underlined.

Amino acid sequences of circulating strains were compared to the vaccine strains in order to highlight functional variation that might potentially impact vaccine efficacy. HA constitutes the receptor-binding and membrane fusion glycoprotein of influenza virus. Alignment of the terminal sialic acid (SA) residues of glycoproteins and glycolipids representing the cellular receptors for influenza virus, which are the targets for neutralizing antibodies, and the conserved N-linked glycosylation sites are shown in **Fig. 3** and **Fig. 4**. The conserved amino acid residues in both H1 and H3 influenza A virus, Tyr(Y)-98, Ser(S)-136, Trp(W)-153, His(H)-183 and Tyr(Y)-195 (numbering according to H3 structure) at the HA receptor-binding site have been described by Skehel and Wiley [14]. These residues were found at the HA receptor binding site of both H1N1 and H3N2. The residues mainly responsible for NeuAcα2,6Gal linkage of H3 are at positions 226 and 228. Amino acids at the terminal SA of all H3 isolates were Ile(I)-226 and Ser(S)-228, similar to those previously reported by Lindstrom et al. [15]. N-linked glycosylation was commonly found in HA. As for HA1 of the H1 subtype, the seven positions of potential N-glycosylation, with a threshold value of >0.5, were predicted (at positions 15, 27, 58, 91, 129, 164 and 290). These sites were found to be conserved among all isolates in this study. Nine potential N-glycosylation sites were predicted in HA1 of the H3 subtype (at positions 8, 22, 38, 63, 126, 133, 165, 246 and 285) with a threshold value of >0.5. These sites were found to be conserved among all isolates obtained during 2006–2009. In comparison with the 2005–2006 strain, the majority of 2006–2007 viruses had lost the predicted glycosylation at position 144; one isolate showed glycosylation at this position (A/Thailand/CU231/2006). The predicted score at position 144 has been under the set threshold value of 0.5 and was therefore not included in this analysis. All isolates from the 2007–2008 and 2009 seasons had lost this position.

The HA1 domain of HA, the major antigenic protein of influenza A viruses, contains all the antigenic sites of HA and is continually under selection pressure driven by the host's immune response. Patterns of antigenic site variations in the HA gene were observed by amino acid alignment. H1N1 antibodies are directed to each of the two strain-specific (Sa and Sb) and common antigenic sites (Ca and Cb) of the virus hemagglutinin [16] (**Fig. 3**). As for H3, the altered amino acids differences were detected at antigenic sites A, B, D and E (**Fig. 4**) Based on amino acid comparison between circulating strains and vaccine strains, the 2005–2006 strains were closely related to the A/Wisconsin67/2005 like lineage vaccine. Although some strains of 2007–2008 had altered amino acid residues at antigenic sites in comparison with the A/Brisbane/10/2007 like lineage, the circulating 2007–2008 strains were closely related to this vaccine subtype. In contrast, the 2009 strains showed variations at 8 positions in HA1 compared to the A/Brisbane/10/2007 like lineage vaccine, including 6 positions at the antigenic site: N/K-144 at site A; N/K-158 and K/N-189 at site B; Q/K-173, R/Q-261 at site E; and A/T-212 at site D. On the other hand, the 2009 strains showed three altered amino acids at the antigenic site when compared to the A/Perth/16/2009-like lineage; S/I-214 at site D; T/A-212 at site D and R/Q-261 at site E.

Alteration at positions prone to induce drug resistance were observed. Oseltamivir resistance associated mutation at position 274 of NA was absent n all H3N2 isolates between 2006 and 2009 and H1 isolates from 2006. In contrast, all H1N1 isolates from 2009 exhibited the oseltamivir resistant amino acid, Y, at position 274 of the NA protein. The S31N substitution in the M2 protein indicates resistance to amantadine, the influenza matrix ion channel inhibitory drug. Five H3N2 isolates from the 2006–2007 seasons (A/Thailand/CU228/2006, A/Thailand/CU231/2006, A/Thailand/CU260/2006, A/Thailand/CU280/2007 and A/Thailand/CU282/2007) displayed Serin (S) at this position indicating amantadine sensitivity. But in the 2007–2009 H3N2 strain, this position had changed to Asparagine (N) implying drug resistance. Only one isolate from 2009 (A/Thailand/CU-H223/2009) displayed Asparagine at this position (amantadine resistance genotype) while all H1N1 isolates showed amantadine sensitive associated genotype.

Some internal proteins harbor the amino acid essential for the HLA epitope. Details concerning alteration of the HLA epitope are shown in supporting information **Table S2**. Most substitutions in the T cell epitope were found in the NP protein of both H1N1 and H3N2 viruses. The substitutions were variable by season or year of collection.

**Figure 5:**
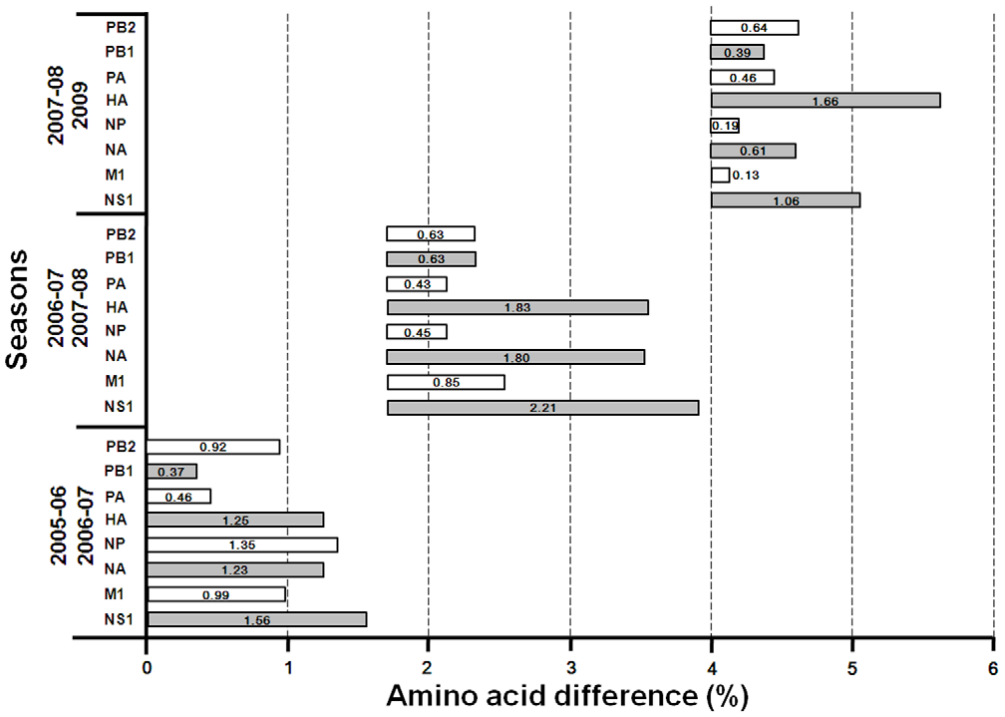
The trend of H3N2 protein distance. Distance means were computed as the arithmetic mean of all pair wise distances between two seasons.

**Figure 6:**
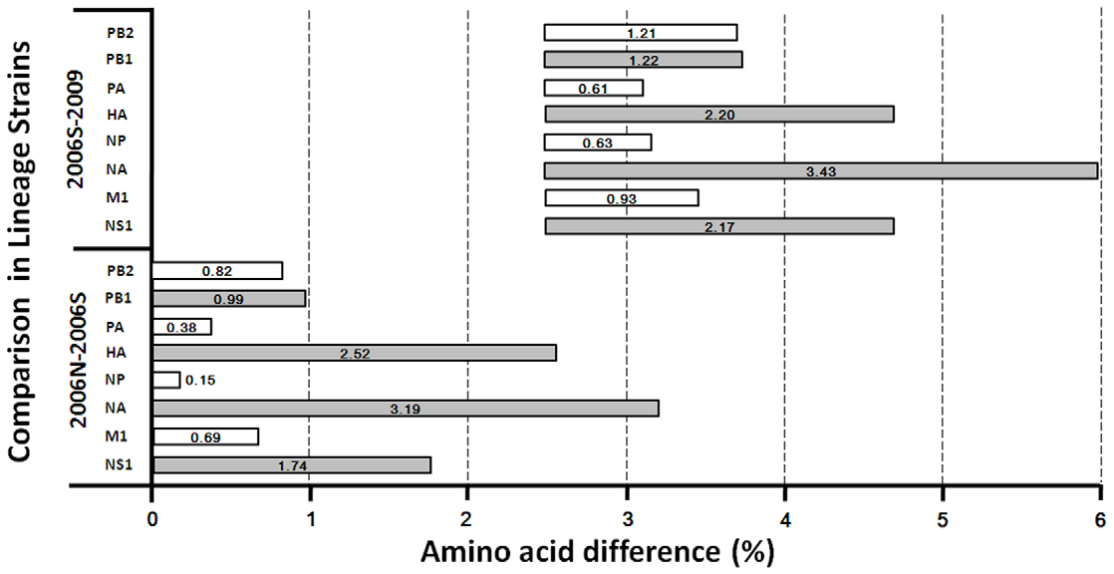
The trend of H1N1 protein distance. Distance means were computed as the arithmetic mean of all pair wise distances between two lineages. 2009 represents the circulating strain in 2009 which belong to A/Brisbane/59/2007-like lineage. 2006S represents the circulating strain in 2006 which belong to A/Solomon Island/3/2006-like lineage. 2006N represents the circulating strain in 2006 which belong to A/New Caledonia/20/1999-like lineage.

Some amino acid of the isolates had undergone variations between two successive seasons demonstrating progressive evolution in each proteins segment. Except for the NS1 protein, HA and NA displayed more variations per season than other proteins **(** **Fig. 5** **)**. The 2009 viruses had fewer variations in the internal proteins compared to the 2007–2008 isolates. On the other hand, the HA of 2009 viruses displayed 1.66% amino acid difference compared to viruses circulating during the previous season. Unfortunately, H1N1 isolates between 2007 and 2008 were not included in this study. Based on amino acid distance comparison of 3 lineage strains, A/NewCaledonia/20/1999 lineage strain, A/Solomon Island/3/2006 lineage strains, and 2009 A/Brisbane/59/2007 lineage strain, was determined in **Fig. 6**. Most amino acid differences between lineages were identified in the surface proteins, HA and NA. As for the internal proteins, NS1 also demonstrated remarkable amino acid variation between lineages. In contrast, NP had undergone the least umber of alterations of all 8 proteins compared.

## Discussion

Each year, WHO recommends the most suitable composition of influenza vaccine strains for the Northern and Southern Hemispheres, based on phylogenetic analyses of HA and antigenic characteristics of circulating viruses. Accordingly, genetic comparison of the HA sequences determined in this study and vaccine strains showed seasonal clusters are closely related to the vaccine strains recommended. H3N2 isolates from the 2005–2006 seasons are closely related to the vaccine strains recommended for 2006–2007 Northern Hemisphere, 2007 Southern Hemisphere and 2007–2008 Northern Hemisphere (A/Wisconsin/67/2005 like lineage). During the 2007–2008 seasons, viruses had drifted towards the sequence of a vaccine strain for 2008 Southern Hemisphere, 2008–2009 Northern Hemisphere and 2009–2010 Northern Hemishphere (A/Brisbane/10/2007 like lineage). Viruses isolated from 2009 are closely associated with the A/Perth/16/2009 (H3N2) like lineage. The A/Perth/16/2009 like lineage represents the most recent vaccine strain for the 2010 Southern Hemisphere influenza season. WHO had recommended the three lineage strains for 2006–2009, the time perod during which the strains had been circulating. The evolutionary rate of H3 is rather high with approximately 1.3–1.8% allelic variation in HA per year.

In contrast, the H1N1 viruses exhibit slow genetic evolutionary rates. The temporal genetic variations we identified were minor. Thus, at the time this study was being performed (2006–2009), only two vaccines were recommended by WHO - A/Brisbane/59/2007 like lineage vaccine and the A/California/7/2009 (H1N1)-like virus [17]. Given the lower rate of change in H1N1 viruses, these vaccines would be expected to confer protection against H1N1 viruses that were recovered from clinical cases in 2009. Although, the rate of genetic change of seasonal H1N1 viruses are not as rapid as that identified in H3N2, further surveillance studies are required.

Sequence analysis of HA showed high variation in HA1, which might be due to its receptor-binding properties and being targeted by neutralizing antibodies since it represents the membrane fusion glycoprotein of influenza virus. The residues within the receptor-binding site are relatively conserved but the residue mainly responsible for NeuAcα2,6Gal linkage specific for the H3 subtype was Ile226 in the place of Leu226 [18]. In H3, amino acid substitutions were detected at three antigenic sites, A, B and E. The antigenic site position preferred for mutation was located at site A. Positions in the H1N1 isolates differentiating them from the A/Solomon Island/3/2006 like lineage and A/Brisbane/59/2007 like lineage were part of the antigenic Sa site. Oligosaccharides in the HA surface proteins might more readily facilitate viral escape from the immune system than single amino acid changes at the antigenic sites. Oligosaccharides may trigger conformational changes in the molecule and mask antigenic sites, which in turn would prevent binding of host antibodies. The predicted N-linked glycosylation at position 144 of the HA antigenic site A has not been observed since the 2006–2007 season. This position may not play any major role in escape from the immune system.

T-cell epitopes in internal proteins of influenza A virus are more conserved than antibody epitopes. The reason for this higher degree of conservation is that 80% of the antibody epitopes are located in the variable glycoproteins HA and NA, while only 40% of the T-cell epitopes are found in these proteins [19]. Most substitutions in regions involved in protective T-cell response were detected in the NP protein, as most T-cell epitopes are defined for the NP protein and this protein constitutes the main target for the host's cytotoxic immune response [20], [21].

During the 2008–2009 season, prevalence of oseltamivir resistance was very high among isolates from over 30 countries [22]. In this study, oseltamivir resistance was detected in H1N1 viruses from 2009 strains. The amantadine drug resistant H3N2 viruses increased during the 2007–2009 season in Thailand, while the most of H1N1was sensitive to amantadine.

Complete genome analysis of human influenza A viruses was necessary to obtain a comprehensive picture of virus evolution. The genetic make-up of influenza A viruses changes every year. Hence, continuous antigen and genome sequence surveillance of influenza A viruses is still a requirement. In this study, our group performed nucleotide sequence comparisons between Thailand strains and vaccine strains provided by WHO. We detected significant numbers ofamino acid substitutions in surface proteins and some in the internal proteins of viruses, which circulated in Thailand over a period of years (2006–2009).

## Materials and Methods

### Clinical samples

All Influenza A virus positive samples obtained during the patients' routine examination or treatment were chosen and stored at −70°C for further analysis. All the patient identifiers were removed from these samples to protect patient confidentiality.Patient consent was practically impossible to obtain. However, permission was granted by the director of the hospital for inclusion of these samples in the study. An ethics committee at the Faculty of Medicine, Chulalongkorn University, Bangkok, Thailand approved all study protocols. An IRB approval was obtained under certification reference number COA No. 287/2008 and IRB No. 391/51, for this study. In addition, all specimens were used exclusively for academic research and the patients were not remunerated. Nasopharyngeal aspirates were randomly selected from the seasonal influenza A positive specimens from the routine clinical service during 2006–2009. All samples were tested for influenza A virus subtypes H1 and H3 using a one-step multiplex real-time RT-PCR as described [7]. Specimens positive for pandemic influenza A H1N1 2009 were excluded from this study.

### RNA extraction and whole genome sequencing

Viral RNA was extracted from 200-µl nasopharyngeal aspiration samples by Real Genomics Viral Nucleic Acid Kit (RBC Bioscience, Taiwan). cDNA was synthesized at 37°C for 3 hours using the M-MLV reverse-transcription system (Promega, Madison, WI) and 1 µM universal primer as described by Hoffmann et al [8]. The whole genome sequences were amplified using the primer sets for human H1N1 and H3N2 influenza A virus (primer sequences are available on request). Briefly, 1 µl of cDNA template was added to the reaction mixture containing 10 µl of 2.5X Eppendorf mastermix (Eppendorf, Hamburg, Germany), 0.5 µM forward and reverse primers and nuclease free water to a final volume of 25 µl. Amplification was performed in a thermal cycler (Eppendorf, Germany) under the following conditions: Denaturation at 94°C for 3 min, followed by 40 amplification cycles consisting of denaturation at 94°C for 30 sec, primer annealing at 50°C for 30 sec (for PB2, PB1, PA genes) and 55°C for 30 sec (for HA, NP, NA, MP, NS genes) followed by extension at 72°C for 90 sec, and concluded by a final extension at 72°C for 7 min. After amplification, electrophoresis was carried out in ethidium bromide containing 2% agarose gels and visualized on a UV trans-illuminator. PCR products were gel-purified using the Perfectprep Gel Cleanup kit (Eppendorf, GmbH, Germany). DNA sequencing (primer sequences on request) was performed by First BASE Laboratories Sdn Bhd (Selangor Darul Ehsan, Malaysia).

### Phylogenetic analysis

Nucleotide sequences were aligned with ClustalX v1.83 [9]. Dendrograms were constructed using the Neighbor-joining (NJ) approach implemented in MEGA 4 [10]. Bootstrap support for tree topologies was accomplished using NJ methods implemented in MEGA with 1,000 iterations. Genetic distances based on NJ phylogenetic trees were calculated applying Kimura's two-parameter method using MEGA 4.

### Prediction of N-Glycosylation

Potential N-glycosylation sites (amino acids Asn-X-Ser/Thr, where X is not Asp or Pro) were predicted using nine artificial neural networks with the NetNGlyc server 1.0 [11]. A threshold value of >0.5 average potential score was set to predict glycosylated sites. The N-Gly-cosite prediction tool at Los Alamos [12] was used to visualize the fraction of isolates possessing certain glycosylation sites along the aligned sequences.

## Supporting Information

Table S1 Accession number of influenza virus that isolated from this study.
(0.07 MB DOC)

Table S2 Amino acid alterations in HLA epitope in internal protein. Alterative amino acids in red and brackets indicate the minor amino acid substitutions.
(0.05 MB DOC)

## References

[1] Webster RG, Bean WJ, Gorman OT, Chambers TM, Kawaoka Y (1992). Evolution and ecology of influenza A viruses.. Microbiol Rev.

[2] Cox NJ, Subbarao K (2000). Global epidemiology of influenza: Past and present.. AnnuRev Med.

[3] Webby RJ, Webster RG (2003). Are we ready for pandemic influenza?. Science.

[4] Peiris JS, Yu WC, Leung CW, Cheung CY, Ng WF (2004). Re-emergence of fatal human influenza A subtype H5N1 disease.. Lancet.

[5] Lin YP, Gregory V, Bennett M, Hay A (2004). Recent changes among human influenza viruses.. Virus Research.

[6] Hampson AW, Potter CW (2002). Influenza virus antigens and antigenic drift..

[7] Suwannakarn K, Payungporn S, Chieochansin T, Samransamruajkit R, Amonsin A (2008). Typing (A/B) and subtyping (H1/H3/H5) of influenza A viruses by multiplex real-time RT-PCR assays.. J Virol Methods.

[8] Hoffmann E, Stech J, Guan Y, Webster RG, Perez DR (2001). Universal primer set for the full-length amplification of all influenza A viruses.. Arch Virol.

[9] Thompson JD, Gibson TJ, Plewniak F, Jeanmougin F, Higgins DG (1997). The CLUSTAL X windows interface: flexible strategies for multiple sequence alignment aided by quality analysis tools.. Nucleic Acids Res.

[10] Tamura K, Dudley J, Nei M, Kumar S (2007). MEGA4: Molecular Evolutionary Genetics Analysis (MEGA) software version 4.0.. Mol Biol Evol.

[11] Gupta R, Jung E, Brunak S (2004). Prediction of N-glycosylation sites in human proteins.. http://www.cbs.dtu.dk/services/NetNGlyc/.

[12] Zhang M, Gaschen B, Blay W, Foley B, Haigwood N (2004). Tracking global patterns of N-linked glycosylation site variation in highly variable viral glycoproteins: HIV, SIV, and HCV envelopes and influenza hemagglutinin.. Glycobiology.

[13] Chutinimitkul S, Chieochansin T, Payungporn S, Samransamruajkit R, Hiranras T (2008). Molecular characterization and phylogenetic analysis of H1N1 and H3N2 human influenza A viruses among infants and children in Thailand.. Virus Res.

[14] Skehel JJ, Wiley DC (2000). Receptor binding and membrane fusion in virus entry: the influenza hemagglutinin.. Annu Rev Biochem.

[15] Lindstrom S, Sugita S, Endo A, Ishida M, Huang P (1996). Evolutionary characterization of recent human H3N2 influenza A isolates from Japan and China: novel changes in the receptor binding domain.. Arch Virol.

[16] Caton AJ, Brownlee GG, Yewdell JW, Gerhard W (1982). The antigenic structure of the influenza virus A/PR/8/34 hemagglutinin (H1 subtype).. Cell.

[17] World Health Organization (2009). Recommended composition of influenza virus vaccines for use in the 2010 southern hemisphere influenza season.. http://www.who.int/csr/disease/influenza/recommendations2010south/en/index.html.

[18] Parrish CR, Kawaoka Y (2005). The origins of new pandemic viruses: the acquisition of new host ranges by canine parvovirus and influenza A viruses.. Annu Rev Microbiol.

[19] Bui HH, Peters B, Assarsson E, Mbawuike I, Sette A (2007). Ab and T cell epitopes of influenza A virus, knowledge and opportunities.. Proc Natl Acad Sci USA.

[20] Townsend AR, Skehel JJ (1984). The influenza A virus nucleoprotein gene controls the induction of both subtype specific and cross-reactive cytotoxic T cells.. J Exp Med.

[21] Winter G, Fields S (1981). The structure of the gene encoding the nucleoprotein of human influenza virus A/PR/8/34.. Virology.

[22] World Health Organization (2009). WHO Epidemic and Pandemic Alert and Response (EPR), Influenza: Influenza A(H1N1) virus resistance to oseltamivir - 2008/2009 influenza season, northern hemisphere.. http://www.who.int/csr/disease/influenza/H1N1webupdate20090318%20ed_ns.pdf.

