# Supplementary material for: Molecular Evolution of Human H1N1 and H3N2 Influenza A Virus in Thailand, 2006–2009
Source: PLoS One. 2010 Mar 16;5(3):e9717. doi: 10.1371/journal.pone.0009717 (PMC2838802; doi:10.1371/journal.pone.0009717)
Supplement: Table S1 — Accession number of influenza virus that isolated from this study. (0.07 MB DOC) [file pone.0009717.s001.doc]

| Strain | Subtype | PB2 | PB1 | PA | HA | NP | NA | M | NS |
| --- | --- | --- | --- | --- | --- | --- | --- | --- | --- |
| A/Thailand/CU23/2006 | H3N2 | FJ912901 | FJ912902 | FJ912903 | EU021266 | FJ912904 | EU021267 | FJ912905 | FJ912906 |
| A/Thailand/CU32/2006 | H1N1 | FJ912907 | FJ912908 | FJ912909 | EU021264 | FJ912910 | EU021265 | FJ912911 | FJ912912 |
| A/Thailand/CU44/2006 | H1N1 | FJ912913 | FJ912914 | FJ912915 | EU021258 | FJ912916 | EU021259 | FJ912917 | FJ912918 |
| A/Thailand/CU46/2006 | H3N2 | FJ912919 | FJ912920 | FJ912921 | EU021268 | FJ912922 | EU021269 | FJ912923 | FJ912924 |
| A/Thailand/CU51/2006 | H1N1 | FJ912925 | FJ912926 | FJ912927 | EU021254 | FJ912928 | EU021255 | FJ912929 | FJ912930 |
| A/Thailand/CU68/2006 | H1N1 | FJ912931 | FJ912932 | FJ912933 | EU021260 | FJ912934 | EU021261 | FJ912935 | FJ912936 |
| A/Thailand/CU88/2006 | H1N1 | FJ912937 | FJ912938 | FJ912939 | EU021253 | FJ912940 | EU021252 | FJ912941 | FJ912942 |
| A/Thailand/CU228/2006 | H3N2 | FJ912943 | FJ912944 | FJ912945 | EU021274 | FJ912946 | EU021275 | FJ912947 | FJ912948 |
| A/Thailand/CU231/2006 | H3N2 | FJ912949 | FJ912950 | FJ912951 | EU021282 | FJ912952 | EU021283 | FJ912953 | FJ912954 |
| A/Thailand/CU260/2006 | H3N2 | FJ912955 | FJ912956 | FJ912957 | EU021280 | FJ912958 | EU021281 | FJ912959 | FJ912960 |
| A/Thailand/CU280/2007 | H3N2 | FJ912961 | FJ912962 | FJ912963 | EU021272 | FJ912964 | EU021273 | FJ912965 | FJ912966 |
| A/Thailand/CU282/2007 | H3N2 | FJ912967 | FJ912968 | FJ912969 | EU021276 | FJ912970 | EU021277 | FJ912971 | FJ912972 |
| A/Thailand/CU1101/2008 | H3N2 | FJ912997 | FJ912998 | FJ912999 | EU625363 | FJ913000 | EU625366 | FJ913001 | FJ913002 |
| A/Thailand/CU1102/2008 | H3N2 | FJ913003 | FJ913004 | FJ913005 | EU625364 | FJ913006 | EU625367 | FJ913007 | FJ913008 |
| A/Thailand/CU1103/2008 | H3N2 | FJ913009 | FJ913010 | FJ913011 | EU625365 | FJ913012 | EU625368 | FJ913013 | FJ913014 |
| A/Thailand/CU356/2008 | H3N2 | FJ912973 | FJ912974 | FJ912975 | FJ912976 | FJ912977 | FJ912978 | FJ912979 | FJ912980 |
| A/Thailand/CU370/2008 | H3N2 | FJ912981 | FJ912982 | FJ912983 | FJ912984 | FJ912985 | FJ912986 | FJ912987 | FJ912988 |
| A/Thailand/CU379/2008 | H3N2 | FJ912989 | FJ91299 | FJ912991 | FJ912992 | FJ912993 | FJ912994 | FJ912995 | FJ912996 |
| A/Thailand/CU-B4/2009 | H3N2 | GQ902790 | GQ902791 | GQ902792 | GQ902793 | GQ902794 | GQ902795 | GQ902796 | GQ902797 |
| A/Thailand/CU-B42/2009 | H1N1 | GQ902798 | GQ902799 | GQ902800 | GQ902801 | GQ902802 | GQ902803 | GQ902804 | GQ902805 |
| A/Thailand/CU-B97/2009 | H1N1 | GU271947 | GU271948 | GU271949 | GU271950 | GU271951 | GU271952 | GU271953 | GU271954 |
| A/Thailand/CU-B106/2009 | H3N2 | GQ983545 | GQ983546 | GQ983547 | GQ983548 | GQ983549 | GQ983550 | GQ983551 | GQ983552 |
| A/Thailand/CU-B110/2009 | H3N2 | GQ902806 | GQ902807 | GQ902808 | GQ902809 | GQ902810 | GQ902811 | GQ902812 | GQ902813 |
| A/Thailand/CU-B267/2009 | H1N1 | GU271955 | GU271956 | GU271957 | GU271958 | GU271959 | GU271960 | GU271961 | GU271962 |
| A/Thailand/CU-B589/2009 | H1N1 | GU183796 | GU183797 | GU183798 | GU183799 | GU183800 | GU183801 | GU183802 | GU183803 |
| A/Thailand/CU-B590/2009 | H3N2 | GQ902814 | GQ902815 | GQ902816 | GQ902817 | GQ902818 | GQ902819 | GQ902820 | GQ902821 |
| A/Thailand/CU-B657/2009 | H3N2 | GQ902822 | GQ902823 | GQ902824 | GQ902825 | GQ902826 | GQ902827 | GQ902828 | GQ902829 |
| A/Thailand/CU-B685/2009 | H1N1 | GU183804 | GU183805 | GU183806 | GU183807 | GU183808 | GU183809 | GU183810 | GU183811 |
| A/Thailand/CU-B1672/2009 | H3N2 | GU271971 | GU271972 | GU271973 | GU271974 | GU271975 | GU271976 | GU271977 | GU271978 |
| A/Thailand/CU-B1697/2009 | H3N2 | GU271979 | GU271980 | GU271981 | GU271982 | GU271983 | GU271984 | GU271985 | GU271986 |
| A/Thailand/CU-H16/2009 | H3N2 | GU271987 | GU271988 | GU271989 | GU271990 | GU271991 | GU271992 | GU271993 | GU271994 |
| A/Thailand/CU-H17/2009 | H1N1 | GQ902830 | GQ902831 | GQ902832 | GQ902833 | GQ902834 | GQ902835 | GQ902836 | GQ902837 |
| A/Thailand/CU-H223/2009 | H1N1 | GU183812 | GU183813 | GU183814 | GU183815 | GU183816 | GU183817 | GU183818 | GU183819 |
| A/Thailand/CU-H565/2009 | H1N1 | GU271963 | GU271964 | GU271965 | GU271966 | GU271967 | GU271968 | GU271969 | GU271970 |

TableS1. Accession number of influenza virus that isolated from this study
