# Supplementary material for: Molecular Evolution of Human H1N1 and H3N2 Influenza A Virus in Thailand, 2006–2009
Source: PLoS One. 2010 Mar 16;5(3):e9717. doi: 10.1371/journal.pone.0009717 (PMC2838802; doi:10.1371/journal.pone.0009717)
Supplement: Table S2 — Amino acid alterations in HLA epitope in internal protein. Alterative amino acids in red and brackets indicate the minor amino acid substitutions. (0.05 MB DOC) [file pone.0009717.s002.doc]

TableS2. Amino acid alterations in HLA epitope in internal protein. Alterative amino acids in red and brackets indicate the minor amino acid substitutions.

| Protein | Epitope | Original Epitope | H1N1 | H3N2 | Note |
| --- | --- | --- | --- | --- | --- |
| PA | HLA-A*0201 PA_225-233_^(*1)^ | CLENFRAYV | CIENFRAYV | CLENFRAYV |  |
|  | HLA-B*8 PA_601-609_ ^(*2)^ | SVKEKDMTK | SVKEKDMTK | SV(I)KEKDMTK | The I602V substitution was found in one H3N2 isolate |
|  |  |  |  |  |  |
| NP | HLA-A*01 NP_44-52_ ^(*3)^ | CTELKLSDY | CTELKLNDY | CTELKLNDY(H) | The original epitope was found in H3N2 isolates from 2005-2006 season. The Y52H substitution was found in H3N2 isolated since 2006-2007 season until 2009 season |
|  | HLA-A*6801 NP _91-99_ ^(*4)^ | KTGGPIYRR | KTGGPIYKR | KTGGPIYRR |  |
|  | HLA-B*1508 NP_103-111_ ^(*5)^ | KWMRELVLY | KWVRELVLY | KWMRELVLY |  |
|  | HLA-B*4002 NP_251-259_ ^(*5)^ | AEIEDLIFL | AEIEDLTFL | AEIEDLIFL |  |
|  | HLA-B*1402 NP_146-154_ ^(*6)^ | TTYQRTRAL | TTYQRTRAL | ATYQRTRAL |  |
|  | HLA-A*1101 NP_188-198_ ^(*1)^ | TMVMELIRMVK | TMVLELIRMIK | TMVMELIRMIK |  |
|  | HLA-DQA1*0501/HLA-DQB1*0201 NP_365-379_ ^(*7)^ | IASNENMDNMGSSTL | IASNENMDAIVSSTL | IASNENMDNMGSS(G)TL | The S377G substitution was found in two H3N2 isolates in 2005-2006 season |
|  | HLA*B27 NP_383-391_ ^(*2)^ | SPYWAIRTR | SRYWAIRTR | SGYWAIRTR |  |
|  | HLA-B*0702/HLA-B*3501 NP_418-426_ ^(*8)^ | LPFEKSTVM | LPFDKA(T)TIM | LPFEKSTIM | The S423T substitution was found in 2006 H1N1 and S423A were found in 2009 viruses |
|  |  |  |  |  |  |
| M1 | HLA-B*39 M1_173-181_ ^(*2)^ | IRHENRMVL | IRHENRMVL | IR(K)HENRMVL | The R174K substitution was found in four H3N2 viruses. |
|  |  |  |  |  |  |
| NS1 | HLA-DR*03 NS1_34-42_ ^(*9)^ | DRLRRDQKS | DRLRR(H)DQKS | DRL(V)RRDQRS | The H39R substitution was found in one 2009 H1N1 virus. |
|  |  |  |  |  | The L36V substitution was found in 4 siolates from 2006-2007 seasons of H3N2 viruses. |
|  | HLA-A*0201 NS1_122-130_ ^(*10)^ | AIMDKNIIL | AIMDKNIIL | AIMEKNIML |  |

*1. Gianfrani C, Oseroff C, Sidney J, Chesnut RW, Sette A. Human memory CTL response specific for influenza A virus is broad and multispecific. Hum Immunol 2000;61:438-52.

*2. Wang M, Lamberth K, Harndahl M, Røder G, Stryhn A, Larsen MV, Nielsen M, Lundegaard C, Tang ST, Dziegiel MH, Rosenkvist J, Pedersen AE, Buus S, Claesson MH, Lund O. CTL epitopes for influenza A including the H5N1 bird flu; genome-, pathogen-, and HLA-wide screening. Vaccine 2007;25:2823-2831.

*3. DiBrino M, Tsuchida T, Turner RV, Parker KC, Coligan JE, Biddison WE. HLA-A1 and HLA-A3 T cell epitopes derived from influenza virus proteins predicted from peptide binding motifs. J Immunol 1993;151:5930-5935.

*4. Guo HC, Jardetzky TS, Garrett TP, Lane WS, Strominger JL, Wiley DC. Different length peptides bind to HLA-Aw68 similarly at their ends but bulge out in the middle. Nature 1992;360:364-366.

*5. Berkhoff EG, Geelhoed-Mieras MM, Fouchier RA, Osterhaus AD, Rimmelzwaan GF. Assessment of the extent of variation in influenza A virus cytotoxic T-lymphocyte epitopes by using virus-specific CD8+ T-cell clones. J Gen Virol 2007;88:530-535.

*6. DiBrino M, Parker KC, Margulies DH, Shiloach J, Turner RV, Biddison WE, Coligan JE. The HLA-B14 peptide binding site can accommodate peptides with different combinations of anchor residues. J Biol Chem 1994;269:32426-32434.

*7. Vartdal F, Johansen BH, Friede T, Thorpe CJ, Stevanović S, Eriksen JE, Sletten K, Thorsby E, Rammensee HG, Sollid LM. The peptide binding motif of the disease associated HLA-DQ (alpha 1* 0501, beta 1* 0201) molecule. Eur J Immunol 1996;26:2764-2772.

*8. Rohrlich PS, Cardinaud S, Firat H, Lamari M, Briand P, Escriou N, Lemonnier FA. HLA-B*0702 transgenic, H-2KbDb double-knockout mice: phenotypical and functional characterization in response to influenza virus. Int Immunol 2003;15:765-72.

*9. Jameson J, Cruz J, Terajima M, Ennis FA. Human CD8+ and CD4+ T lymphocyte memory to influenza A viruses of swine and avian species. J Immunol 1999;162:7578-7583.

*10. DiBrino M, Parker KC, Margulies DH, Shiloach J, Turner RV, Biddison WE, Coligan JE. Identification of the peptide binding motif for HLA-B44, one of the most common HLA-B alleles in the Caucasian population. Biochemistry 1995;34:10130-10138.
